# The Indoor Environment at the University Equestrian Facility in the Autumn Semester: A Case Study

**DOI:** 10.3390/ani15223322

**Published:** 2025-11-18

**Authors:** Pavel Kic, Marie Wohlmuthová

**Affiliations:** 1 Department of Technological Equipment of Buildings, Faculty of Engineering, Czech University of Life Sciences Prague, 16521 Prague, Czech Republic; kic@tf.czu.cz; 2 Department of Mathematics and Physics, Faculty of Engineering, Czech University of Life Sciences Prague, 16521 Prague, Czech Republic

**Keywords:** airborne dust, CO_2_, health, horse welfare, measurement, noise, relative humidity, riding arena, stable, temperature

## Abstract

This article aims to show some of the factors that influence the internal environment in a horse stable under the operating conditions of a university farm during the autumn semester. The conditions inside the stable were favorable in terms of temperature and air humidity as well as CO_2_ concentration during this period. According to anonymous statements of 94 respondents (workers, students, and members of the equestrian club), 83% of them suffer from occasional respiratory problems, which could be contributed to by the deterioration of the indoor environment, especially dustiness. Analysis of the measured dust concentration values showed that the largest share of the dust concentration was made by large particles above 10 μm. There was no difference between the average concentration of PM_10_ dust particles (877 μg·m^−3^) during bedding with straw or a mixture of sawdust and shavings. The average concentration of PM_10_ particles during the cleaning of the corridor was also high (185.9 ± 105.4 μg·m^−3^). On the contrary, the average concentration of PM_2.5_ particles during the cleaning of the corridor (173.1 ± 50.3 μg·m^−3^) was higher than during bedding with straw (54.4 ± 34.9 μg·m^−3^) or a mixture of sawdust and shavings (69.2 ± 22.1 μg·m^−3^). The average concentration of dust during the movement of horses in the outdoor riding arena was within the recommended limit values for outdoor limits of PM_10_ and PM_2.5_. The average sound pressure level L_pAi_ = 47.0 ± 8.7 dB fluctuated during the day, but the horses were not disturbed during rest and sleep, even though this equestrian facility is located near a commercial airport.

## Simple Summary

This article evaluates the basic parameters of the indoor environment, such as temperature and humidity, CO_2_ concentration, airborne dust concentration, and noise in the operating conditions of a university horse farm during the autumn semester. The measurements showed that the conditions inside the stable were favorable in terms of temperature and humidity as well as CO_2_ concentration during this period. However, the air quality is worsened by the high concentration of dust, especially during cleaning, which can have a bad effect not only on the stabled horses but also on the workers, students, and members of the riding club. The airborne dust was characterized by a high proportion of the largest dust particles. Analysis of the measurement results showed that there was no difference between the average concentration of PM_10_ dust particles during bedding with straw or a mixture of sawdust and shavings. Noise measurements showed that the average sound pressure level did not exceed the recommended limits, even though this equestrian facility is located near a commercial airport.

## 1. Introduction

Although a large part of the world’s horse population is currently used for agricultural work, the breeding and use of horses represents an important part of cultural, sporting, and recreational life, especially in Western countries in the 21st century.

In the Czech Republic, horse breeding has a long tradition and is increasingly popular. According to [1], the total number of horses in the Czech Republic increased to 106,643 registered animals in 2023 compared to 88,347 registered in 2014. Horses are used not only in elite sports—horse racing, dressage, and show jumping—but also increasingly in hobby and recreational activities, such as horse riding, agrotourism, or hippotherapy. This wide scope is also reflected in the academic environment. The Czech University of Life Sciences in Prague (CULS), specifically the Faculty of Agrobiology, Food and Natural Resources, offers a bachelor’s program in “Horse Breeding”, which integrates theoretical and practical education over three years [2]. Students gain knowledge of training, breeding, housing, nutrition, ethology, and veterinary care as well as complete professional practice during the semester.

A prerequisite for the successful provision of education and other sports or recreational activities related to horse breeding is the provision of suitable stable accommodation for horses [3,4,5] and the provision of suitable equipment and an environment for students and other people actively involved in the operation of the stable [6,7]. The priority for the construction of a horse stable is to provide suitable accommodation for horses at a level corresponding to the requirements of animal welfare [8,9,10,11]. However, attention should also be paid to the conditions for staff (caretakers, teachers, trainers) and students.

According to surveys concerned with a variety of topics, including the environment in the equestrian facilities, problems of a stuffy indoor environment are usually manifested by air pollution by airborne dust and higher air humidity, which is usually caused by insufficient ventilation [12,13,14]. The indoor environment in livestock barns is also affected by changes in the external climate year-round, even in winter [15]. As expected, due to year-round higher temperatures in Central Europe, temperatures in the stables are higher, but due to the different structural features of the buildings, the internal conditions are different. According to [16,17], the structural and technical type and orientation in relation to the cardinal points have a significant impact on the temperature conditions in the stable. The health of horses is benefited by the opportunity to spend more time outdoors on pasture [18]. The issue of noise in horse stables also needs attention, as horses are very sensitive to various sounds (similarly to other farm animals) [19,20,21,22,23] and changes in sound pressure levels [24].

A crucial factor in terms of the welfare and health of horses is the quality of the indoor environment of stables. An optimal microclimate includes suitable air temperature, relative humidity, low concentration of airborne dust, and controlled carbon dioxide concentration [6,25,26,27]. Horses tolerate a wide range of temperatures well, but the optimal temperature range in a stable is between 15 and 20 °C in the summer. Lower temperatures (up to −6 °C) are not problematic with sufficient ventilation and a dry environment; on the contrary, temperatures above 25 °C can negatively affect the thermoregulation and well-being of the horse. The optimal temperatures in a stable may vary slightly depending on the type of horse. According to [6], 18 °C in winter and 18–22 °C in summer are sufficient for sport horses. Relative humidity should be 50–75%. Values below 40% increase the risk of drying out the mucous membranes and irritation of the respiratory tract, while higher humidity (>80%) promotes the growth of mold and bacteria, thereby increasing the microbial load of the environment. According to [25], the relative humidity should not exceed 85%. Carbon dioxide is an indicator of the quality of ventilation. The maximum concentration of CO_2_ in the stable should not exceed 3000 ppm [28], with ideal values below 2000 ppm. Higher concentrations indicate insufficient ventilation and are often associated with the accumulation of other pollutants.

Achieving and maintaining an optimal microclimate in the stable is crucial not only for the health of horses but also for their performance and overall well-being. Proper ventilation, quality bedding, and regular maintenance of stable spaces fundamentally affect the parameters of the internal environment. Poor conditions in the stable negatively affect the immunity, vitality, and willingness to work of horses [29,30]. Physiological parameters such as heart rate and locomotor activity are important for assessing animal welfare [31]. Different housing conditions and seasons are reflected in hematological parameters and leukocytes [32]. Higher values of cortisol, total protein, and α1- and α2-globulins were observed in July in carriage horses compared to May and June. These changes were probably caused by the increase in the temperature humidity index (THI) [33] values, which showed moderate stress in June and high stress in July [34]. In sport horses, changes in leukocytes and lymphocytes are caused not only by physical activity but also by the time of day [35]. According to [36], hematological parameters show different circadian rhythmic behavior in horses housed loose in an individual box and horses housed in a paddock during the four seasons. Horse welfare can be threatened by many factors, and exposure to adverse conditions can lead to stressful conditions [37,38,39,40]. It is therefore necessary to continue to investigate the relationship between the state of well-being of horses in their environment and different riding situations [4].

The amount of airborne dust in the indoor environment is also related to the cleaning of the stable, bedding, and ventilation. The concentration of airborne dust has a direct impact on the incidence of respiratory diseases [41]. According to current studies, the concentration of inhaled PM_10_ particles should not exceed 150 µg/m^3^, but ideally it should remain below 100 µg/m^3^. The composition of the airborne dust is also an important factor, both in terms of the size of the dust particles and the content of endotoxins and molds. Horses are susceptible to respiratory diseases related to dustiness [42]. Adequate ventilation, together with the quality of bedding and feed, are important factors affecting the welfare of horses. Dusty materials handled in horse stables, especially feed and bedding, are a source of dust. Airborne dust threatens the health of horses and can cause various respiratory diseases [26,27] which impair their training capabilities [43].

The issue of airborne dust and the situation in terms of dust concentration in various facilities for breeding livestock has received considerable attention [44,45,46]. Airborne dust is a carrier and spreader of microbes [47,48,49,50,51,52,53,54,55,56,57,58]. Therefore, airborne dust is a problem for the health of people (caretakers, riders, etc.). With the growing interest in horse breeding, the number of workers, caretakers, and other people moving around in the environment of stables and horse breeding areas is also increasing. However, the number of people who show deterioration in their health and various forms of allergy to the dusty environment in these horse breeding areas is also increasing [56,59,60,61,62,63,64].

Research has shown that some allergic conditions such as hay fever, eczema, and asthma are partly influenced by hormones and brain cells that are released into the bloodstream in response to stress [65]. Similarly to humans, stress is also important for animals [66] and has been studied in horses [49,52,66,67,68,69]. However, dust is also a health problem for horses. The impact of airborne dust on horse health has received considerable attention [26,53,54,63,70,71,72,73,74,75,76,77], and it is important for breeders and technicians involved in the design of stables and technological equipment for horse farms to monitor the sources of dust. In addition to stabled animals, bedding is a major source of dust particles [49,51,58,77,78,79,80,81,82,83,84,85,86,87,88,89,90,91,92,93,94].

The bedding material is one of the important factors influencing the welfare of stabled animals. Various materials are used, both organic and inorganic, with their advantages and drawbacks. The basic task of bedding is to absorb urine and moisture; at the same time, it should provide comfort to horses when lying down, it should not slip when lying down or standing up, and it should provide thermal insulation [89,90]. Bedding should also be safe for horses from a health point of view, i.e., it should be free of dust particles, free of mold spores or other pathogens, and non-irritating [91]. However, it should be safe from a health point of view not only for horses, but also for grooms and riders, or people who regularly spend a long time in the stable.

Each type of bedding has its advantages, and it depends on the stable management which one they prefer. The ideal bedding should be easily available, harmless to health, dust-free and easy to handle, easy to store, easily compostable, and also cheap [92]. One of the most commonly used beddings is straw. Its advantages are its thermal insulation properties; it is soft and therefore pleasant for horses. It is easily available and compostable. Fresh manure from straw bedding is suitable for the production of biogas or biomethane [93,94]. Its disadvantage is its dustiness; it often contains mold spores [92]. Long straws, in particular, are less able to absorb urine. It has higher storage requirements [89] and higher losses because horses consume it.

Another frequently used type of bedding is wood sawdust and shavings, or a mixture of them. The advantages are high absorbency [95], low losses, easy handling and storage of packaged products, and minimal fungal spores [96]. The disadvantages of packaged products are a higher price, higher dustiness, poorer storage stability of unpackaged products, and possible impurities (varnishes, nails, etc.). Compared to straw, they have worse thermal insulation properties.

In some stables, especially for racehorses abroad, shredded paper is used [90,92]. Its advantages are minimal dustiness and high absorbency; it is a good insulator. The disadvantages are higher consumption (it shrinks a lot), poorer handling, and easy mold growth. Sand is often used, especially for shelters in grazing stables. Its advantages are low dustiness; the disadvantages are different quality and possible sharpness/roughness. It freezes in winter and does not insulate [90]. Other options include peat moss, flax and hemp bast, cardboard, and rubber matting [92]. Last but not least, special products used mainly in modern sports stables should be mentioned. This category includes, among others, granulated wheat straw, pressed wood shavings, pressed ground wheat straw, etc. The advantages of these products are high absorbency, low losses, easy handling, and storage due to the fact that they are packaged products. Another great advantage is that they are dust-free and free of fungal spores [90].

However, dustiness is also influenced by feed and feed handling, as well as the design and layout of the stable. The issue of size and solution of the method of housing in terms of space utilization and design of the stable is also addressed in some works [43,49,77,82,83,84,85,87,88,97,98,99,100]. Dust in horse stables and the impact of housing is monitored in [82,88,101,102].

From the perspective of the overall assessment of the well-being of stabled horses, the design and construction of the stable in given climatic conditions have a very significant influence [43,51,57,80,102,103]. The influence of the ventilation system and ventilation on the indoor environment, including airborne dust concentration, is significantly manifested [85,101].

Some research also pays attention to the cleanliness of the air and dust in other rooms and places on horse farms. In addition to staying in the stable, horses, riders, and grooms are also in training halls (riding halls) [53,57,62,83,104,105] and in the outdoor environment (in the paddock) [53,83,102]. Operating conditions and airborne dust occurrence are thus more significantly influenced by the season and weather [53,55,73,83,103,105,106].

Particle size analysis and various measurement methods are being validated for practical implementation based on scientific procedures and operating conditions [50,57,82,83,102,103]. Considerable attention is also paid to the quality and properties of the bedding materials [107,108].

There are no prescribed limit values for assessing dust concentration in horse stables, therefore it is appropriate to use the limit value for outdoor airborne dust [109], which according to [110] is 50 μg·m^−3^ for 24 h PM_10_, 40 μg·m^−3^ for the annual limit value and 25 μg·m^−3^ for PM_2.5_.

This article aims to expand and deepen information on microclimatic conditions in equestrian facilities operated within the CULS in Prague in the autumn semester. The research was focused not only on assessing technological and operational influences on air temperature and humidity, CO_2_ concentration, and noise in the stable, but above all, more attention was paid to the problems of airborne dust. Determination of dust particle concentrations and analysis of PM composition in terms of dust fractions PM_10_, PM_4_, PM_2.5_, and PM_1_ show the need for further research in this area to ensure welfare both for horses and staff.

## 2. Materials and Methods

### 2.1. Description of the Farm

This research work and measurements were carried out on a horse farm situated at Dvorská 1, on the outskirts of Prague, in the central part of the Czech Republic. This farm consists of different facilities. The most important facilities for our measurements were a stable, an indoor riding arena, and an outdoor riding arena, described in more detail in [109].

In the stable, students interact with representatives of various horse breeds, including thoroughbreds that have retired from racing. The most famous representative of the racehorses stabled here was the white mare Sixteen [111], who spent her retirement here from 2012 to 2020. This mare won the Velká pardubická steeplechase (a 6900 m course with 31 obstacles, it is one of the hardest steeplechases in Europe) twice, in 2007 and 2008. In 2008, she and jockey Bartoš set the fastest time in the history of the Velká pardubická, 8:58 min, which has so far been beaten only in 2015 [112]. Daily programs in the autumn semester of the academic year during a working day or non-working day are presented in Table 1 and Table 2.

The operating conditions are influenced by the overall situation and the number of students at the university and their interest in horse breeding. Average data from recent years show that 200 to 300 students complete a seven-day or two-week internship in this equestrian facility every year. The equestrian facility also hosts courses taught by the Faculty of Agrobiology, Food and Natural Resources of the CULS in Prague. The most important courses in terms of the number of students are:

- Horse Breeding and Riding,
- Ethics and Welfare of Horse Breeding,
- Equine-assisted Therapy/activities,
- Buildings for Horse Husbandry,
- Equestrian Sport and Horse Riding,
- Training of Horse and Rider.

**Figure 1:**
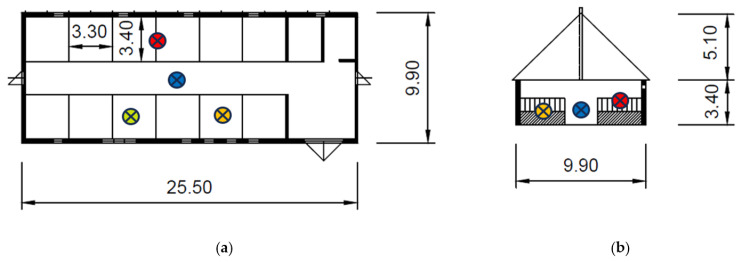
The stable: (**a**) the stable floor plan. (**b**) The cross-section (space for stabling horses, without facilities for students). 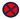
—position of long-term measurement devices, 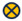
—position of airborne dust measurement in the box with straw bedding, 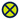
—position of airborne dust measurement in the box with a mixture of sawdust and shavings, 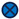
—position of airborne dust measurement in the central corridor.

**Figure 2:**
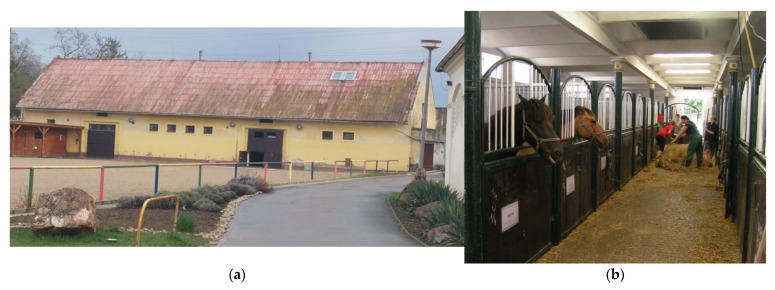
The stable: (**a**) the north side of the stable. (**b**) Internal central corridor during cleaning.

The masonry building of the stable has a gable tin roof. Details were described in [109]. The location of the measurement points is shown in Figure 1. Figure 2 shows the exterior and interior views of the stable. At the time of the research measurements, 12 horses were housed in the stable.

Available material is used for bedding in the stalls, which is alternately short-cut straw or a mixture of sawdust and shavings in a ratio of 1:1. Straw consumption is approximately 10 kg/horse/day, and sawdust/shavings consumption is approximately 11–12 kg/horse/day.

**Figure 3:**
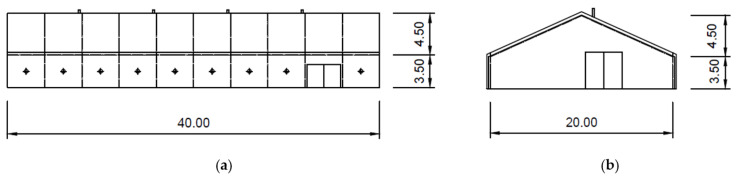
The indoor riding arena: (**a**) side and (**b**) front view.

**Figure 4:**
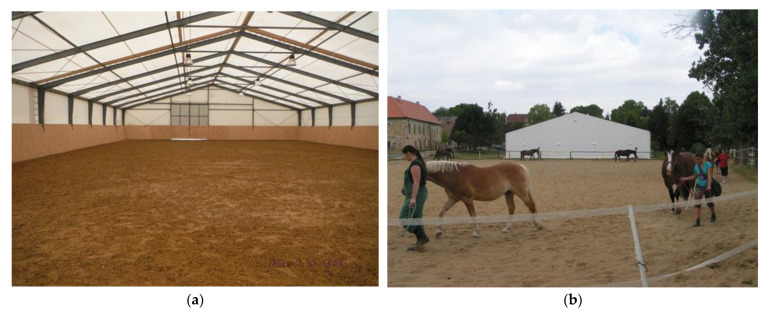

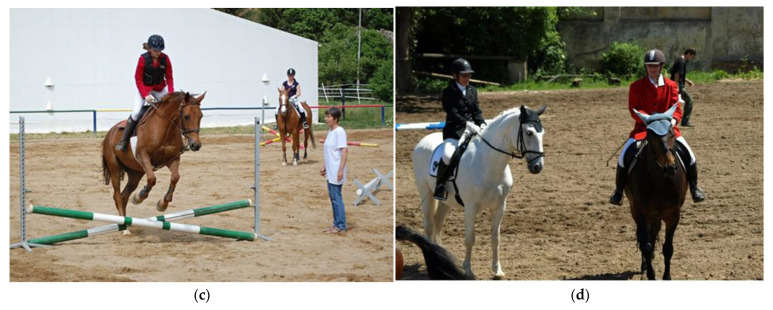
The riding arenas: (**a**) the interior of the indoor riding arena with a modified floor surface. (**b**,**c**) Students training in an outdoor riding arena. An indoor riding arena is in the background. (**d**) Riders before the competition.

The indoor riding arena (Figure 3 and Figure 4a) was described in detail in [109]. The outdoor riding arena (Figure 4b–d) has dimensions of 48 m by 32 m, and the surface is sandy.

### 2.2. Data Acquisition

Measurement of basic parameters was based on the methodology described in [109]. A summary of the measuring devices and sensors used in this research is presented in Table 3. The following data loggers were used: ZTH 65, where the manufacturer and supplier of the ZTH 65 data loggers is COMET SYSTEM, s.r.o., Czech Republic, Almemo 2590-9 and 2690 data loggers with the corresponding sensors (FHA 646, FYA 600, BEHA Unitest 93411 D) are from Ahlborn (Ahlborn Mess- und Regelungstechnik GmbH, 306 Eichenfeldstraße 1, 83607 Holzkirchen, Germany) [113], Dust-Track™ II Aerosol Monitor 8530 is manufactured by TSI Incorporated, 500 Cardigan Road Shoreview, MN 55126 US, data loggers MN 55126 and Kern-440-35N are produced by KERN & SOHN GmbH, Ziegelei 1, 72336 Balingen-Frommern, Germany.

The measurements were mainly focused on determining the concentration of airborne dust in the air with the aim of showing whether there is any difference between the dust concentration during various work activities in the stable and also to show what this concentration is in terms of the size of individual size fractions of airborne dust particles.

The measurement was performed during the following activities:

- Cleaning and removal of manure from boxes.
- Bedding the boxes with straw.
- Bedding the boxes with sawdust and wood shavings.
- Sweeping and cleaning of the central corridor.
- Empty indoor arena without horses, slightly wet surface covered by sand and a mixture with a small quantity of geotextile; summer conditions.
- Horses in external riding paddock–arena (trotting horses around).

**Figure 5:**
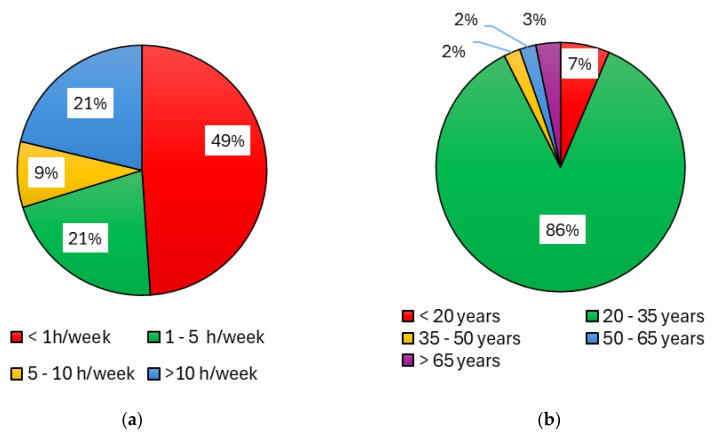
Percentage structure of the questionnaire respondent group depending on: (**a**) time of exposure to the stable and riding arenas environment; (**b**) age structure of the respondent group.

**Figure 6:**
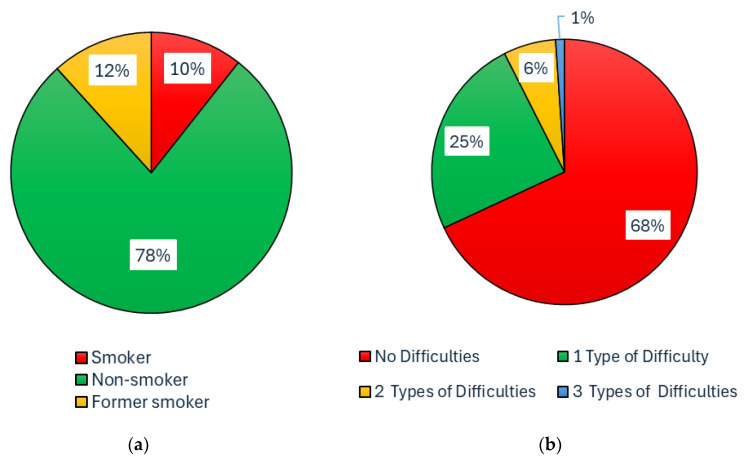
Possible previous influence on the occurrence of subjective respiratory problems—Percentage structure of the questionnaire respondent group depending on: (**a**) Smoking; (**b**) Previous medical history connected with respiratory diseases—difficulties such as allergies, asthma, chronic obstructive pulmonary disease, and their combinations.

Given the importance of the investigated impact of dust on the health of workers, an anonymous survey “Questionnaire: dust in stables/riding stables—impact on health” [114] was also prepared. In this questionnaire, people staying in the stable and its surroundings (workers, students, and members of the riding club) expressed their subjective experiences with the impact of dust in the environment in the horse stable or riding arena and their personal health/respiratory/allergic problems. A total of 94 respondents participated in this anonymous questionnaire survey [114] during the semester. The percentage representation of the various categories is shown in Figure 5 and Figure 6.

Respondents answered the following questions:

1. Age;
2. Gender;
3. How many hours a week do you usually spend in the stables/riding hall (less than 1 h per week, 1–5 h per week, 5–10 h per week, more than 10 h per week);
4. Have you felt repeatedly (more than 3 times a year) any of the following difficulties over the past 2 years (you can mark more possibilities: dry/irritating cough, moist/productive cough, (allergic) rhinitis, shortness of breath/difficulty breathing, none of the above);
5. Smoking (non-smoker, former smoker, smoker-up to 10 cigarettes a day, heavy smoker—10 or more cigarettes a day); and
6. Previous medical history (more possibilities: asthma, allergies, chronic obstructive pulmonary disease, none of the above).

For the purposes of further processing, respondents were divided into four groups (Figure 5a) according to how many hours per week they usually spend in the stable, riding hall and outdoor riding arena. The groups were called No Exposure (NoEx; less than 1 h per week), Low Exposure (LoEx; 1–5 h per week), Medium Exposure (MedEx; 5–10 h per week), and High Exposure (HiEx; more than 10 h per week). Figure 5b and Figure 6a,b show the composition of the research sample of 94 respondents in terms of age, relationship to smoking, and previous medical history. Short-term measurements were collected at the beginning of the autumn semester (5th to 11th October). The operation in the stable is different during working days, when there is teaching of students, and on weekend days.

For the evaluation, according to [6,25,28,29] the following boundary conditions were taken into account. According to [25,29], the optimal temperature in a riding stable in summer is between 15 and 20 °C, the maximum temperature t_imax_ = 25 °C, in winter the temperature should not fall below t_imin_ = 6 °C. The optimal relative humidity RH_io_ should be in the range of 60–80%, the maximum relative humidity RH_imax_ = 85%. In [6], the optimal temperature range in summer is 18–22 °C, in winter 6–15 °C for draft horses, and 10–18 °C for sport horses. The optimal relative humidity RH_io_ is in the range of 50–75%, and the maximum permissible relative humidity RH_imax_ = 85%.

In [28] the following were listed: lower limit of the temperature optimum t_io_ = 10 °C, the lowest permissible temperature t_imin_ = 5 °C, upper limit of optimal relative humidity RH_io_ = 75%, the highest permissible relative humidity RH_imax_ = 85%, the limit of the optimal range of CO_2_ concentration in a stable for horses K_uio_ = 2500 ppm, and the permissible concentration of CO_2_ in a stable for horses K_uimax_ = 3500 ppm.

### 2.3. Analysis of Basic Properties of Bedding Materials

Two types of bedding materials are used in the investigated stable: a mixture of sawdust and shavings in a ratio of 1:1 or short-cut wheat straw. Since the bedding material used can partially influence the dust concentration inside the stable, measurements of the basic properties of the bedding materials under handling conditions during bedding were carried out, which are bulk density and moisture.

Bulk density was determined by gravimetric measurement using measuring containers and a digital laboratory balance KERN-440-35N according to Equation (1).

(1) $$\rho_{V}=\frac{M_{N}}{V_{i}}=\frac{M_{B}-M_{C}}{V_{i}}$$

where $\rho_{V}$—bulk density, kg·m^−3^; *M_N_*—net weight of measured bedding material, kg; *M_B_*—weight of a vessel with measured bedding material, kg; *M_C_*—weight of a vessel, kg; *V_i_*—inner volume of a vessel for measuring bedding material, m^3^.

The determination of litter moisture was carried out according to the methodology described in the literature [115,116].

Water content, wet basis w is the ratio of the mass of water $m_{W}$ contained in a solid to the mass of the moist solid *m =* $m_{S}$ *+* $m_{W}$, expressed in Equation (2):

(2) $$w=\frac{m_{W}}{m}100$$

where w—water content, wet basis, %; $m_{W}$—mass of water, g; $m_{S}$—mass of dry basis, g.

### 2.4. Data Processing

Measurement results from the Dust-Track™ II Aerosol Monitor 8530 instrument were obtained in Excel files. The measured values from the Almemo measuring instruments were first processed using the AMR-Control software version 5.14.0.212. The measured data sets were processed using Microsoft® Excel® 2019 MSO (Version 2402 Build 16.0.17328.20346) and the selected results were analyzed using the statistical software TIBCO SW Data Science Workbench Statistica version 6, ANOVA and Tukey’s HSD (Honestly Significant Difference) test. Graphs expressing the results were processed in MS Excel.

## 3. Results

The measured results are processed gradually in several parts, according to the parameters being measured and the measurement time.

### 3.1. Long-Term Registration Measurement of the Basic Parameters of the Indoor Environment

Long-term recording of temperature and relative humidity, CO_2_ concentration, and noise was carried out for 7 days.

#### 3.1.1. Results of Measurements of the Indoor Environment in the Stable

The results of external conditions measurement and the indoor environment measurement are presented in Table 4.

#### 3.1.2. Analysis of the Indoor Environment in the Stable During a Working Day

**Figure 7:**
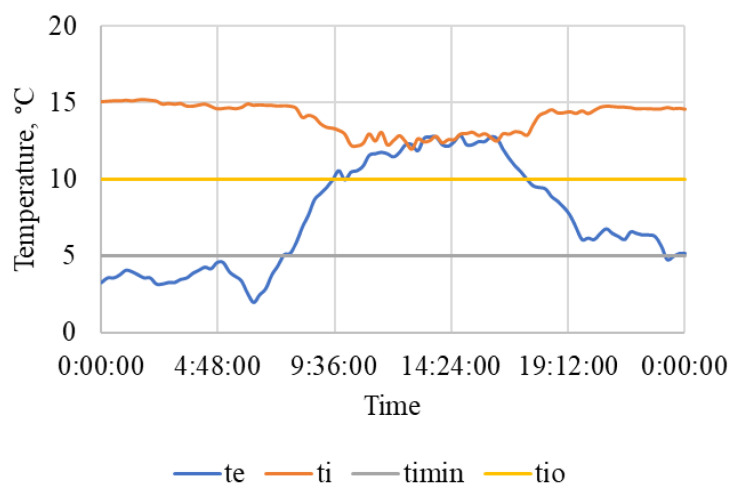
The course of air temperature (°C) outside t_e_ and in the stable t_i_ during a working day. t_e_—outside air temperature, t_i_—air temperature in the stable, t_imin_—minimum permissible air temperature, t_io_—lower limit of optimal air temperature for horses, time (h:min:s).

**Figure 8:**
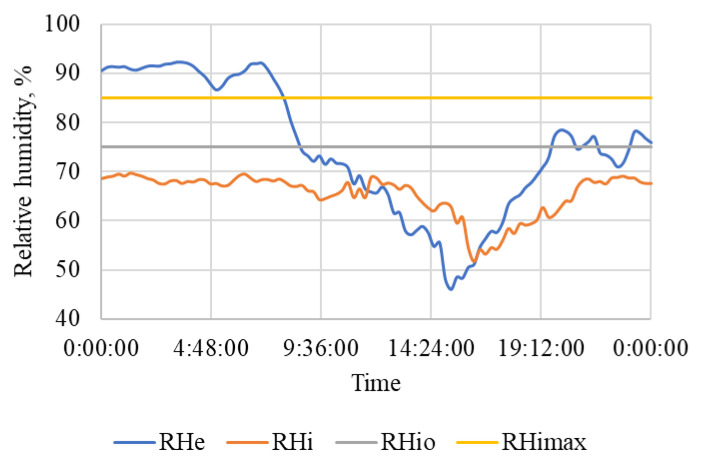
Course of air relative humidity (%) outside and in the stable during a working day. RH_e_—outdoor air humidity, RH_i_—indoor air humidity, RH_io_—upper limit of optimal air humidity, RH_imax_—the highest permissible relative air humidity, time (h:min:s).

**Figure 9:**
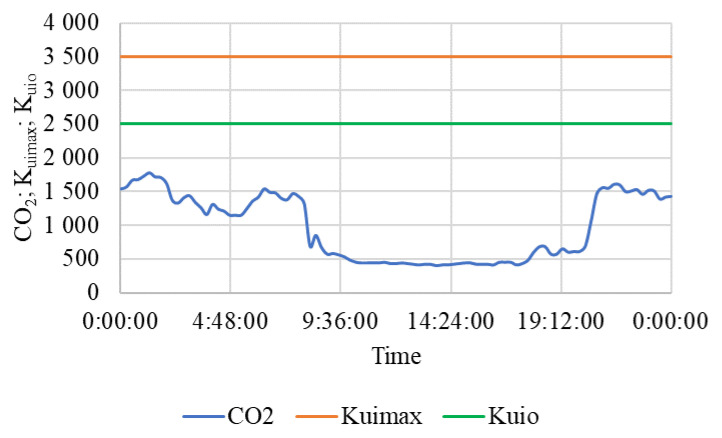
Course of concentration CO_2_ (ppm) in the stable during a working day. CO_2_—concentration of CO_2_, K_uimax_—the highest permissible limit of CO_2_ concentration, K_uio_—the highest limit of the optimal CO_2_ concentration in the stable, time (h:min:s).

The results of measurement on a selected working day of the beginning of the autumn period are summarized in Table 5 and also shown in Figure 7, Figure 8 and Figure 9. The measured values show a significant difference compared to the results obtained in winter [109].

Figure 7 shows that the internal air temperature t_i_ was maintained within the optimum temperature range t_i_ = 14.0 ± 1 °C and never dropped below the lower limit of the optimum of 10 °C, even though the external air temperature dropped below 5 °C at night. On the contrary, the figure shows a drop in air temperature during the warmest period of the day, when there are various activities in the stable from approximately 8 to 18:30, and the horses are also moving outside the stable in the outdoor riding arena or in the paddock. At that time, in the stable there is intensive natural ventilation through open windows and other ventilation openings.

Figure 8 shows the course of outdoor RH_e_ and indoor air humidity RH_i_ during this workday. The figure also shows the highest permissible relative air humidity, RH_imax_ = 85% and the upper limit of the optimal air humidity, RH_io_ = 75% according to [28].

The outdoor relative humidity varied significantly during the day, which is also related to the significant change in the outdoor air temperature (Figure 7). The indoor relative humidity was constantly maintained below 70% even at night, with high outdoor relative humidity and lower ventilation intensity.

The average CO_2_ concentration was 966.4 ± 490.7 ppm. An overview of the CO_2_ concentration inside the stable during one typical working day, and the highest permissible limit K_uimax_ = 3500 ppm, and the highest limit of the optimal CO_2_ concentration in the stable K_uio_ = 2500 ppm are shown in Figure 9.

From Figure 9, it can be seen that during the whole day, even at night, with lower ventilation intensity, the CO_2_ concentration did not reach the highest limit of the optimal concentration of 2500 ppm in the stable. During the time the horses were outside the stable and with the ventilation holes, windows, and doors wide open, the CO_2_ concentration (Figure 9) and the relative humidity of the indoor air (Figure 8) decreased significantly.

#### 3.1.3. Analysis of the Indoor Environment in the Stable During a Non-Working Day

**Figure 10:**
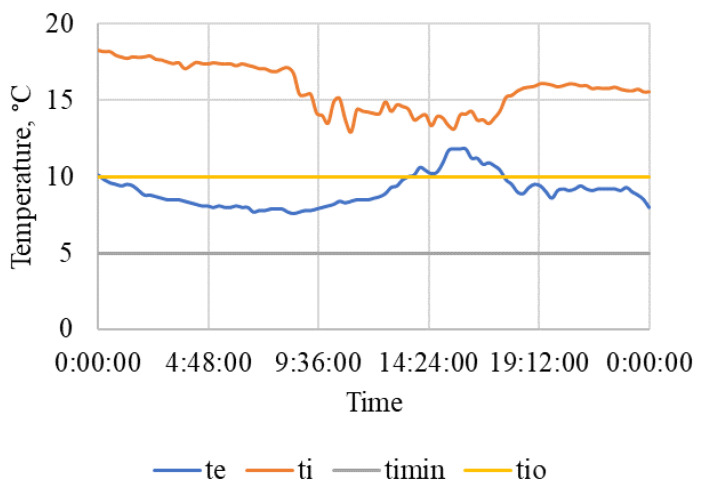
The course of air temperature (°C) outside and in the stable during a non-working day. t_e_—outside air temperature, t_i_—air temperature in the stable, t_imin_—minimum permissible air temperature, t_io_—lower limit of optimal air temperature for horses, time (h:min:s).

**Figure 11:**
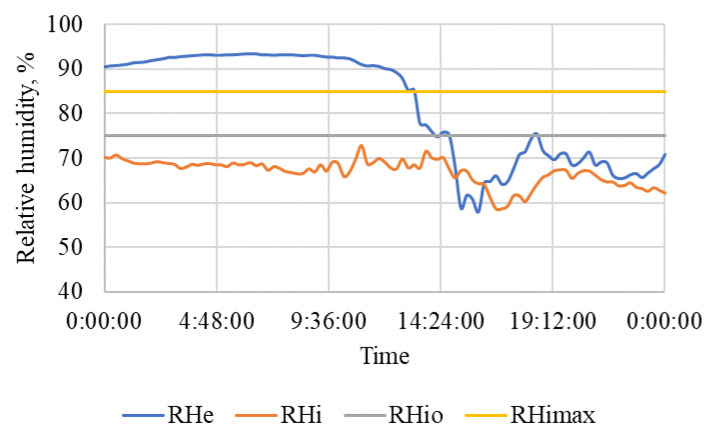
The course of air relative humidity (%) outside and in the stable during a non-working day. RH_e_—outdoor air humidity, RH_i_—indoor air humidity, RH_io_—upper limit of optimal air humidity, RH_imax_—the highest permissible relative air humidity, time (h:min:s).

**Figure 12:**
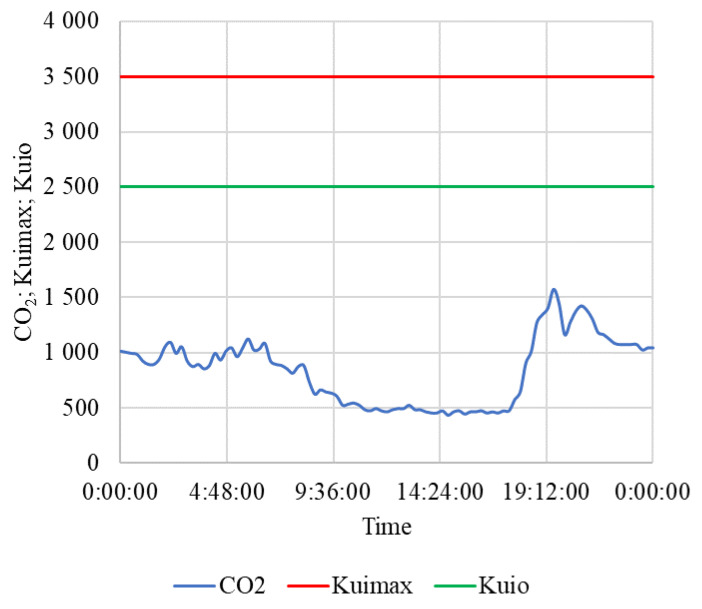
The course of concentration CO_2_ (ppm) in the stable during a non-working day. CO_2_—concentration of CO_2_, K_uimax_—the highest permissible limit of CO_2_ concentration, K_uio_—the highest limit of the optimal CO_2_ concentration in the stable, time (h:min:s).

The results of measurement on a selected non-working day are in Table 6, and the courses are in Figure 10, Figure 11 and Figure 12. The weather was relatively favorable, which was reflected in the favorable conditions inside the stable.

The average outdoor temperature this non-working day was 9.1 ± 1.0 °C, and the indoor air temperature was maintained within the optimal temperature range t_i_ = 15.8 ± 1.5 °C, and the corresponding relative humidity was favorable throughout the day and did not exceed the optimal limit values (Figure 10 and Figure 11).

The situation inside the stable was similarly favorable in terms of CO_2_ concentration (Figure 12). Thanks to sufficient ventilation, the average concentration of CO_2_ = 836.2 ± 303.3 ppm was low throughout the day.

### 3.2. Measurement of Airborne Dust

#### 3.2.1. Results of Measurements of Bulk Density and Moisture of Bedding Materials

**Figure 13:**
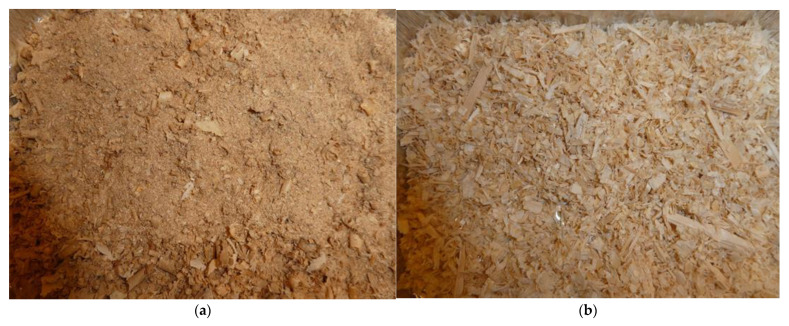

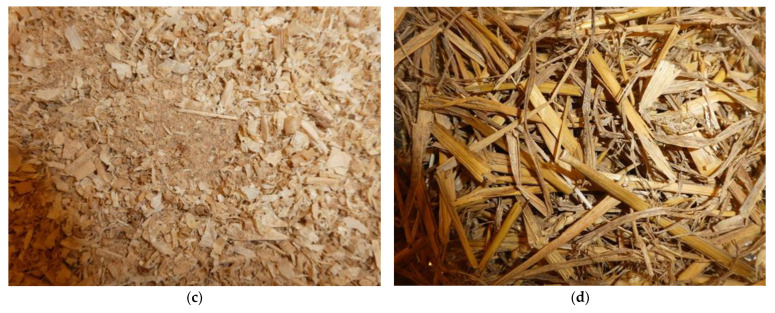
Bedding material before drying: (**a**) sawdust; (**b**) shavings; (**c**) mixture of sawdust and shavings 1:1; and (**d**) straw.

**Figure 14:**
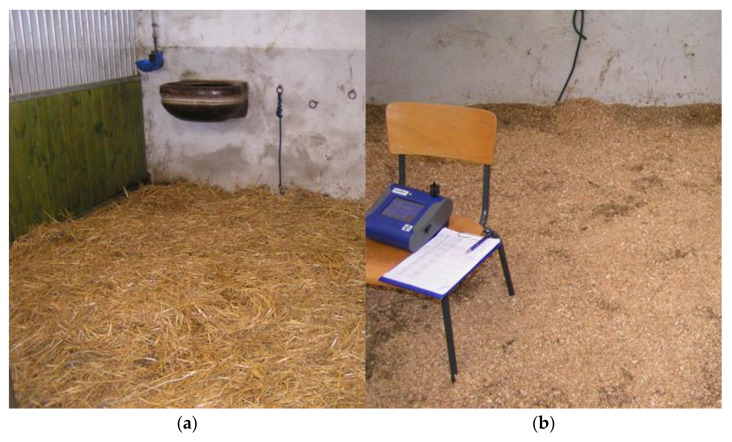
Inside the box with bedding: (**a**) straw. (**b**) Mixture of sawdust and shavings.

For bedding in individual horse boxes, short-cut straw or a mixture of sawdust and shavings is usually used. The mixture is prepared by stable workers in a large container by mixing the two components in a volume ratio of approximately 1:1. Photographs of fresh bedding material before drying are shown in Figure 13. The use of straw and mixture of sawdust with shavings in boxes is shown in Figure 14.

The results of measuring the bulk density and moisture of straw and a mixture of sawdust and shavings are presented in Table 7.

#### 3.2.2. Results of the Questionnaire Survey

**Figure 15:**
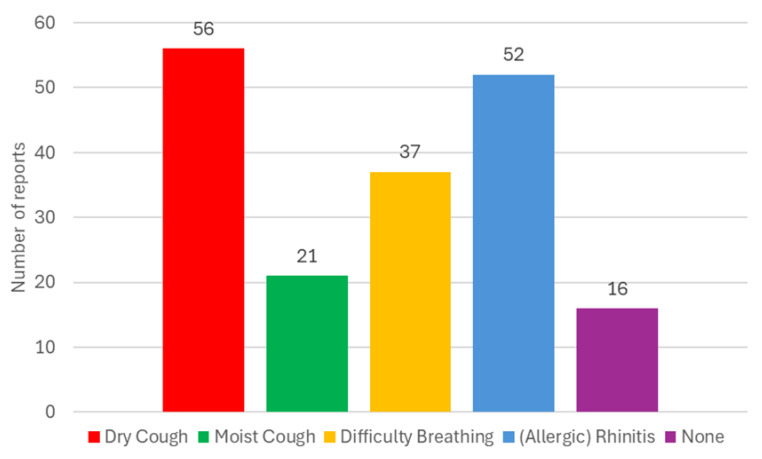
Type and number of reported respiratory problems according to participants’ subjective assessment (94 participants, more options possible).

The summary results of the questionnaire survey regarding reported subjective (i.e., in this text it means subjectively felt by the respondent, not necessarily confirmed by a doctor’s diagnosis) difficulties are shown in Figure 15; respondents could choose from multiple options.

As already mentioned, for a more detailed examination, respondents were divided into four groups: NoEx, LoEx, MedEx, and HiEx, according to the reported average weekly length of stay in the stable, indoor riding arena, and outdoor riding arena. Table 8 shows the number and percentage of smokers (up to 10 cigarettes per day), non-smokers, and former smokers in the NoEx, LoEx, MedEx, and HiEx groups. Among all 94 respondents, there were no heavy smokers (10 or more cigarettes per day). Table 9 shows the number of diseases in the previous medical history within each group, including the percentage representation. Table 10 shows how many respondents in each group have a specific respiratory disease (asthma, allergy, chronic obstructive pulmonary disease) in their previous medical history.

**Figure 16:**
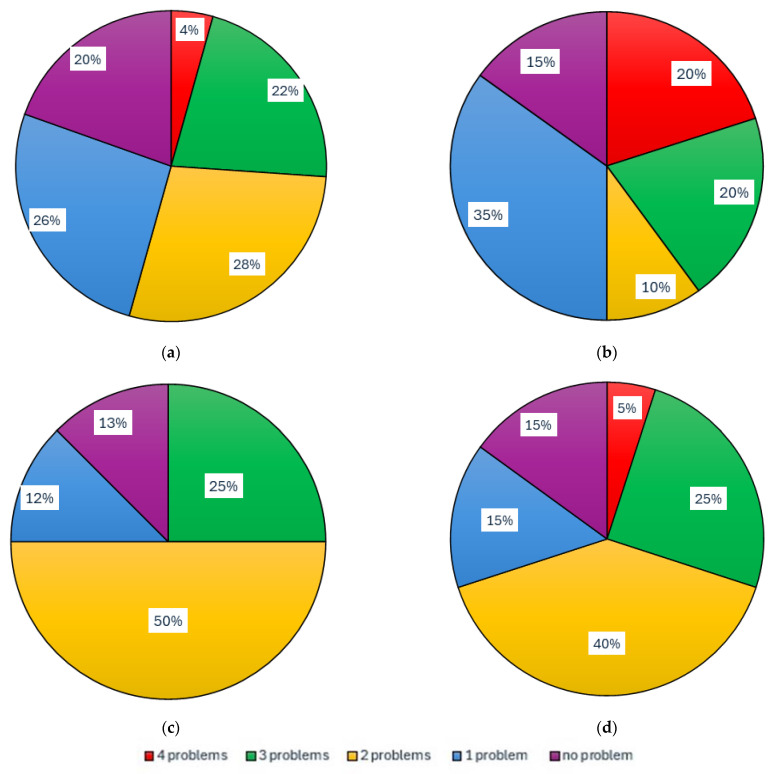
Percentage of respondents depending on the number (0, 1, 2, 3, 4) of reported subjective respiratory problems in groups according to the duration of stay in the equestrian facilities environment: (**a**) NoEx—less than 1 h per week; (**b**) LoEx—1 to 5 h per week; (**c**) MedEx—5 to 10 h per week; (**d**) HiEx—more than 10 h per week.

Reported subjective respiratory problems were recorded in individual groups, as shown in Table 11. The percentage representation for the individual groups NoEx, LoEx, MedEx, and HiEx is shown in Figure 16. The representation of specific respiratory problems (allergic rhinitis, dry cough, moist cough, difficulty breathing, none of the above; multiple choices possible) in the NoEx, LoEx, MedEx, and HiEx groups is shown in Table 12.

#### 3.2.3. Results of Airborne Dust Measurement

Total dust mass concentration TDC (μg·m^−3^) and concentrations of fractions (μg·m^−3^) PM_10_, PM_4_, PM_2.5_, and PM_1_ in the stable during various activities and in the indoor arena without horses and in the outdoor arena with horses are shown in Table 13.

**Figure 17:**
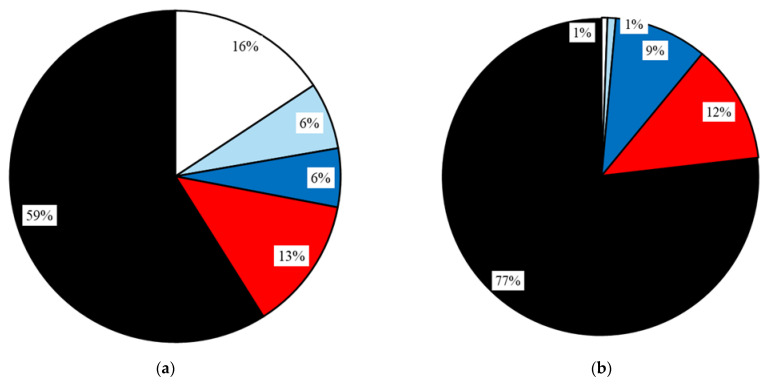

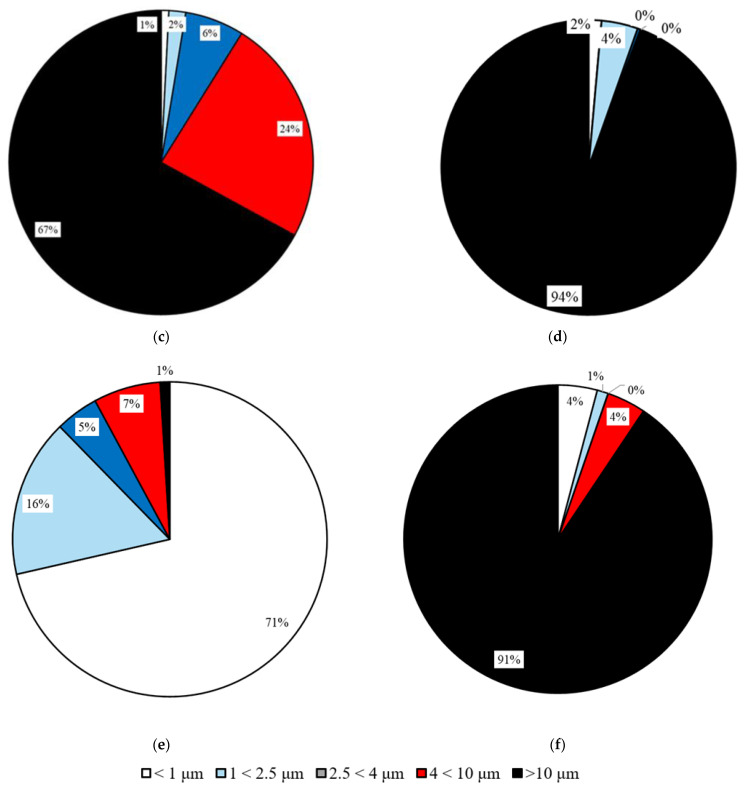
The share of the size distribution of dust particle fractions PM inside the buildings in the range above 10 μm, from 4 μm to 10 μm, from 2.5 μm to 4 μm, from 1 μm to 2.5 μm, and smaller than 1 μm is in percentage of the total airborne dust concentrations in the examined barns during different activities: (**a**) cleaning and removal of manure from boxes; (**b**) bedding the boxes with straw; (**c**) bedding the boxes with saw dust and wood shavings; (**d**) sweeping and cleaning of central corridor; (**e**) empty indoor arena without horses; and (**f**) horses in external riding paddock–arena.

The share of fractions of airborne dust particles in the air in individual buildings during different activities is shown in Figure 17.

### 3.3. Measurement of the Noise Level in the Stable

The results of the noise registration measurement for the entire 7-day measurement period are shown in Table 14. In the following section, an analysis of operational influences affecting noise during the working day and non-working day in the examined periods is provided.

**Figure 18:**
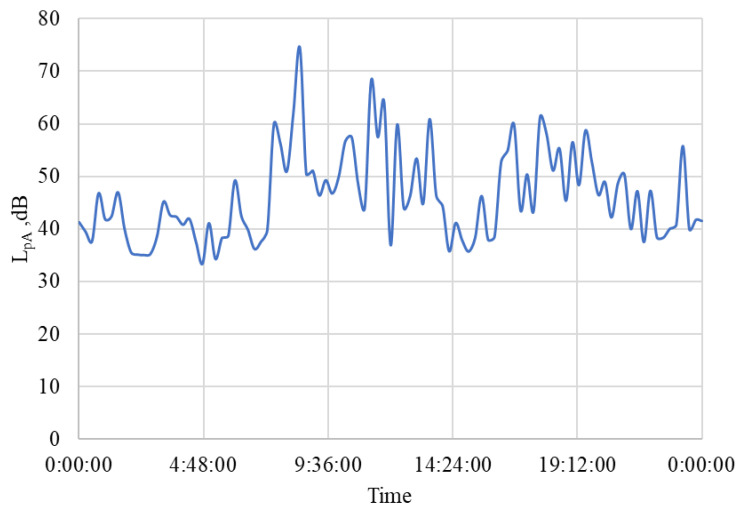
The course of noise measurement in the stable during working day. L_pA_—sound pressure level (dB), time (hour:min:sec).

The results of the recorded noise measurement during the working day are summarized in Table 14, and the course over 24 h is shown in the graph in Figure 18. The noisiest activity was the horses moving to and from the paddock (L_pA_ = 63.0 ± 9.8 dB). Overall, the conditions in the stable can be assessed as quiet, which is also consistent with the conditions observed in the winter period [109].

**Figure 19:**
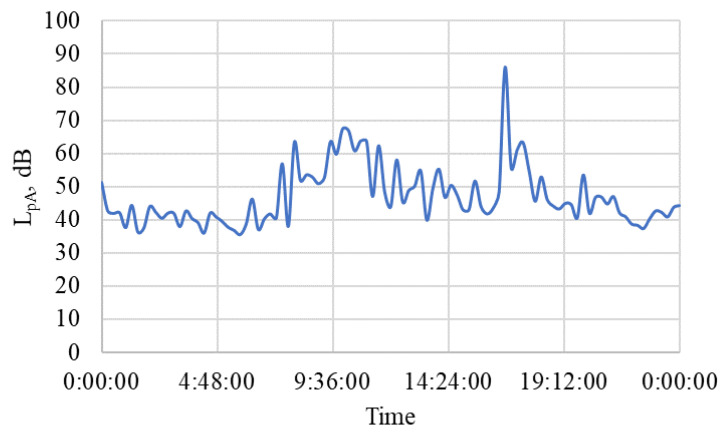
The course of noise measurement in the stable during a non-working day. L_pA_—sound pressure level (dB), time (hour:min:sec).

The results of the recorded noise measurement during the non-working day are summarized in Table 15, and the course over 24 h is shown in the graph in Figure 19. Operating conditions during a non-working day in the winter period do not differ much from a working day, except that there are no regular classes. More significant noise was briefly observed during the activities of the Equestrian club (maximum L_pA_ = 86.0 dB), which could have been caused by random higher noise penetrating the stable from outside, e.g., a plane passing by from a nearby airport.

## 4. Discussion

The aim of the research was to collect quantitative data under real operating conditions in equestrian facilities and, based on their analysis, to contribute new knowledge to deepen information about the quality of the indoor environment in these facilities. Animal welfare and protection of the working environment are coming to the forefront of society’s interest. The use of modern measuring technology allows for more detailed monitoring and analysis of microclimatic conditions. Determination of dust particle concentrations and analysis of PM composition together with feedback from workers, students, and people in equestrian facilities was the main subject of this research.

The research on which this article is based took place in a relatively favorable period of early autumn compared to the previous article [109], which describes the conditions in the winter period. For 7 days of measurement, the average outdoor air temperature was 10.0 ± 4.9 °C with an average relative humidity of 71.7 ± 13.7% and an average wind speed of 2.2 ± 1.3 m·s^−1^. This created favorable conditions in terms of the thermal state in the stable, as shown in the results in Table 4. It also provides the prerequisites for creating a suitable microclimate for stabled horses, workers, students, and sports riders in accordance with the requirements, for example, according to [3,4,5,6]. The spatial design and equipment of the stable also meet the basic requirements for animal welfare according to [7,8,9,12,13,14] so that teaching and other sports activities can take place.

On both working days and non-working days, the stable and other related facilities are very actively used and the microclimatic conditions, including noise (Table 5 and Table 6 and Figure 7, Figure 8, Figure 9, Figure 10, Figure 11 and Figure 12), did not exceed the recommended requirements [6,7,8,9,12,13,14]. An important place in the operation of these activities [3,4,5,6] is also played by good equipment in terms of conditions for the horses to stay outside the stable [12,13] in adjacent land used as a grazing paddock, an open riding arena, and a covered riding arena for bad weather conditions (Figure 1, Figure 2, Figure 3 and Figure 4).

The issue of airborne dust concentration is problematic and important from the point of view of health and working conditions [42,56,57,58,59,60,61,62,63,64,65], which concerns not only stables but also indoor and outdoor riding arenas [12,13,14,117]. The focus of research and measurements has therefore also been on this issue.

For this reason, an anonymous questionnaire survey was prepared [114] (Figure 5 and Figure 6). The results of this anonymous survey are presented in Table 8, Table 9, Table 10, Table 11 and Table 12 and Figure 15 and Figure 16. These results show that 83% of respondents occasionally suffer from subjective respiratory problems, which could be contributed to by exposure to dusty environments. Most frequently reported complaints were dry/irritating cough (60% of all respondents, with the highest percentage in the HiEx group, 75% of the group) and (allergic) rhinitis (55% of all respondents, with the highest percentage in the MedEx group, 63% of the group). Combinations of two or more types of subjective symptoms were reported by 45% of all respondents, most frequently by respondents from the MedEx group (75% of the group) and HiEx group (70% of the group). In total, 17% of all respondents did not report any subjective respiratory problems. The lowest percentage of subjective respiratory problems was recorded in the NoEx group, where 20% of respondents did not report any subjective problems. Consistent with the conclusions of [41], these results suggest that regular repeated visits to equestrian facilities and thus higher exposure of respondents to dust particles may lead to a higher incidence of respiratory problems, especially dry/irritating cough. The data obtained does not allow for clear conclusions to be drawn about the impact of smoking, partly because there were no heavy smokers (10 or more cigarettes per day) among the respondents. However, the high percentage of smokers (20%) and former smokers (20%) in the LoEx group, combined with the fact that 20% of respondents in this group report four subjective respiratory problems, indicates this possibility. A possible influence of previous medical history should be investigated in more detail on a larger number of respondents, as 68% of all respondents did not report any previous medical history, with only two respondents (i.e., 2% of all) reporting chronic obstructive pulmonary disease and only eight respondents (i.e., 9% of all) reporting asthma.

According to [49,51,58,77,88,91,92], bedding is a significant source of dust particles. Therefore, related measurements were carried out to determine the basic properties of the bedding materials used (Table 7 and Figure 13). Both types of bedding materials are used similarly to other farms [89,90,91,92,93,94,95,96], and the current availability of their acquisition is usually decisive.

The measured results of TDC and concentrations of fractions (Table 13 and Figure 17) show that the airborne dust concentrations differed significantly in many cases. The highest average TDC = 3786.7 μg·m^−3^ was measured during the handling of straw and its bedding in the stalls. Bedding with a mixture of sawdust and shavings had a lower effect, but TDC = 2663.5 μg·m^−3^ was still very high. Sweeping and cleaning the central corridor also significantly increased TDC = 3186.2 μg·m^−3^. Shoveling horse manure from the stalls and clearing outside the stable did not have such a significant effect on TDC. TDC = 20.3 μg·m^−3^ in an empty winter arena with a floor surface treated by moistening was very low; higher TDC = 476 μg·m^−3^ in a summer outdoor arena in which horses and riders moved.

High TDC values were also reflected in high PM values. High average PM_10_ values for straw bedding (877.2 μg·m^−3^) and sawdust and shavings bedding (877.5 μg·m^−3^) were not statistically significantly different.

In accordance with the evaluation according to the methodology presented in [109], and according to [110], the PM_10_ limit value in 24 h is 50 µg·m^−3^, the 1-year limit value is 40 µg·m^−3^ and 1-year limit value PM_2.5_ is 25 µg·m^−3^. According to [118], particulate matter (PM_10_ and PM_2.5_) are responsible for significant negative effects on human health, adverse health conditions, and increased mortality. In order to reduce harmful effects on human health, there will be gradual changes in the assessment of airborne dust in terms of the concentration of PM_10_ and PM_2.5_ dust particles. Limit values for the concentration of particulate matter (PM_10_ and PM_2.5_) should be set. For comparison with the resulting PM_10_ and PM_2.5_ values measured in this research and presented in Table 13, only selected data from this directive for 2026 and 2030 are presented in Table 16.

According to the measurement results shown in Table 13, during the time of bedding in the boxes and during cleaning of the center corridor, all prescribed values of concentration of PM_10_ and PM_2.5_ dust particles are exceeded several times. Since the measurements also included the determination of PM_4_ and PM_1_ values, it was possible to analyze the composition of dust particles and the proportion of individual size fractions in more detail (Figure 17) than required by [118]. Large particles, larger than 10 μm, prevailed at this time. The proportion of the smallest PM_1_ particles was not large at the time of these work activities, but the concentration of these smallest particles is significant, and for future research, attention should also be paid to their influence and effect on the health of people or horses.

According to this assessment, the average PM_10_ values found during bedding handling and bedding box placement are many times higher. PM_10_ was also very high during sweeping and cleaning of the central corridor (PM_10_ = 185.9 µg·m^−3^).

The PM_4_ value = 416 µg·m^−3^ was also highest during straw bedding; however, the PM_4_ values were also statistically significantly lower during bedding with a mixture of sawdust and shavings and during cleaning of the central corridor.

If we consider the concentration of smaller particles, the limit value PM_2.5_ 25 µg·m^−3^ was exceeded most during sweeping and cleaning of the central corridor, PM_2.5_ = 173.1 µg·m^−3^. PM_2.5_ was also higher when bedding was manipulated, but the difference between using straw (54.4 µg·m^−3^) or sawdust and shavings (69.2 µg·m^−3^) was not statistically significant.

The concentration of the smallest dust particles, PM_1_ = 45.2 µg·m^−3^, was highest when sweeping and cleaning the central corridor. The difference between bedding with straw (PM_1_ = 22.6 µg·m^−3^) or a mixture of sawdust and shavings (PM_1_ = 21.6 µg·m^−3^) was not statistically significant.

According to the results of PM fraction measurements in a large-scale study [50], the concentration of bacterial aerosol inside stables was many times higher than outside. In stables, before application of bedding, the average concentrations of PM_10_ ranged from 117 to 177 µg·m^−3^, PM_4_ from 115 to 155 µg·m^−3^, PM_2.5_ from 117 to 143 µg·m^−3^, and PM_1_ from 107 to 135 µg·m^−3^. The concentration of airborne dust fractions increased significantly with bedding manipulation.

The PM_10_ and PM_2.5_ values measured in this study and shown in Table 8 were lower than in [50] for all activities during the measurements in the stable and in the indoor riding arena. It is possible that this was influenced by the more favorable weather and more intensive ventilation during this study.

The lowest dust particle concentrations when evaluated according to all measurement methods and relevant criteria were in the empty indoor riding arena during operational standstill, without horses and without movement. It can be assumed that the moistened floor had a great influence on this, which is also in line with the results according to [104,105].

Publications [88,98] report a reduction in the concentration of respirable dust particles (from 86.7 µg·m^−3^ to 26.0 µg·m^−3^) by changing the bedding from straw to shavings and using silage instead of hay and straw as feed, or by treating the hay with moisture. Attempts to reduce dust by replacing straw with shavings (38.07% reduction in average PM_10_ concentration) and straw pellets (51.12% reduction) have reached similar conclusions [81,83].

The percentage of size fractions expressed in Figure 17 shows that this proportion is highly variable. The largest size fraction during all cleaning activities and bedding inside the stable is the largest particles above 10 μm.

During cleaning and removal of manure from boxes, the proportion of the largest particles (over 10 μm) was 59%, but the proportion of the smallest particles (16%) smaller than 1 μm was also significant. Bedding the boxes with straw caused a high proportion (77%) of the largest particles over 10 μm. When bedding the boxes with sawdust and wood shavings, the proportion of the largest particles (over 10 μm) was 67% and the proportion of particles from 4 μm to 10 μm was quite high (24%). The proportion of particles over 10 μm was 94% during sweeping and cleaning of central corridor, particles from 1 to 2.5 μm 4%, and particles smaller than 1 μm only 2%. The proportion of the remaining particles was negligible.

When horses were moving in an external riding paddock–arena, particles above 10 μm were 91%. On the contrary, in the indoor riding arena, which is used in the summer only in very adverse weather conditions, the smallest particles, smaller than 1 μm, had the largest percentage (71%). The proportion of the largest particles was only 1%. The overall low concentration of dust particles and the small proportion of the largest particles can be explained by the high surface humidity of the floor, which is in accordance with the findings [76].

The location of the stable and other related facilities (riding arenas and paddock) is not far from the airport (7.5 km). According to information available in [119,120], the farm area is in a zone with a noise indicator for overall noise nuisance L_den_ = L_dvn_ = 50–60 dB (L_dvn_ = noise indicator for day-evening-night). The registered noise measurement in the stable revealed an average sound pressure level of L_pAi_ = 47.0 ± 8.7 dB for the measured period (Table 4), which is lower than what was measured during the research in the winter period [109]. The occasional higher values measured during the working day L_pAi_ = 74.5 dB or on the day of rest L_pAi_ = 86.0 dB are probably caused by the sudden movements and sounds of the stabled horses, or the source may be the take-off of a commercial aircraft from a nearby airport and the penetration of noise through open windows and doors. The maximum prescribed noise level of 85 dB [22,23] was exceeded exceptionally, only by short-term impulse effects. It is important that during the night hours, the average sound pressure level fluctuated around L_pAi_ = 42 dB, so that the horses were not disturbed during rest and sleep.

Based on the research carried out, it would be appropriate to further focus on monitoring the micro-climatic conditions in equestrian facilities in the summer. So far, winter and autumn periods have been monitored; the summer period could worsen the welfare of horses in terms of the risk of heat stress. It would also be interesting to monitor how long airborne dust and individual dust fractions remain in the air and to determine its microbiological contamination in the indoor environment of the stable and indoor riding arena.

## 5. Conclusions

The research activity verified the microclimatic conditions in the stable for horses in the operating conditions of the university equestrian facilities during the autumn semester. The results of this research showed that the conditions inside the stable were favorable in terms of temperature and air humidity during this period. Thanks to sufficient ventilation, the CO_2_ concentration was also low. However, the air quality was worsened by high airborne dust content, especially during the period of handling bedding and cleaning the corridor in the stable.

Based on an anonymous questionnaire survey of 94 people staying in and around the stable (employees, students, and members of the riding club), it was found that regular repeated stays in equestrian facilities and thus higher exposure of respondents to dust particles can lead to a higher incidence of respiratory problems, especially dry/irritating coughs. Combinations of two or more subjective respiratory problems were reported by the highest percentage (75%) of respondents who spend 5 to 10 h per week in equestrian facilities, and by 70% of respondents who spend more than 10 h per week in equestrian facilities.

Analysis of the measured airborne dust concentration values showed that large particles above 10 μm had the largest share of the dust concentration. There was no difference between the average concentration of PM_10_ dust particles (877 µg·m^−3^) during straw bedding or a mixture of sawdust and shavings. The average concentration of PM_10_ particles during corridor cleaning was also very high (185.9 ± 105.4 µg·m^−3^). On the contrary, the average concentration of PM_2.5_ particles during corridor cleaning (173.1 ± 50.3 µg·m^−3^) was higher than during straw bedding (54.4 ± 34.9 µg·m^−3^) or a mixture of sawdust and shavings (69.2 ± 22.1 µg·m^−3^). The average airborne dust concentration during the movement of horses in the outdoor riding arena was within the recommended limit values for outdoor PM_10_ and PM_2.5_ limits. The average sound pressure level L_pAi_ = 47.0 ± 8.7 dB fluctuated during the day, but the horses were not disturbed during rest and sleep, even though this equestrian facility is located near a commercial airport.

## Figures and Tables

**Table 1:** Daily program in the autumn semester of the academic year—working day.

Activity	Time [from–to]	Duration [min]
Feeding (hay, oats, mown grass)	8:00–8:30	30
Taking the horses to paddocks	8:30–9:00	30
Boxes cleaning, bedding	9:00–11:00	120
Washing troughs and waterers	11:00–11:30	30
Dispensing oats into the troughs	11:30–12:00	30
Returning the horses to the stable	12:30–13:00	30
Feeding the horses	13:00–13:15	15
Students’ activities (more noise)	13:00–15:45	165
Equestrian club activities	15:45–18:00	135
Feeding (hay, oats)	18:00–18:30	30
Locking up, staff leaving	18:30–18:45	15

**Table 2:** Daily program in the autumn semester of the academic year—non-working day.

Activity	Time [from–to]	Duration [min]
Feeding (hay, oats, mown grass)	8:00–8:30	30
Taking the horses to paddocks	8:30–9:00	30
Boxes cleaning, bedding	9:00–11:00	120
Washing troughs and waterers	11:00–11:30	30
Dispensing oats into the troughs	11:30–12:00	30
Returning the horses to the stable	14:00–14:15	15
Feeding the horses	14:15–14:30	15
Equestrian activities (as agreed)	14:30–18:00	210
Feeding (hay, oats)	18:00–18:30	30
Locking up, staff leaving	18:30–18:45	15

**Table 3:** The summary of the measuring devices and sensors.

Instrument	Measured Parameter	Range	Sensitivity	Accuracy
ZTH 65	Air temperature	−30 to +70 °C	0.1 °C	±0.4 °C
ZTH 65	Air relative humidity	5 to 95%	0.1%	±2.5%
Almemo 2590-9	Data logger	9 inputs for sensors	-	-
Almemo 2690	Data logger	5 inputs for sensors	-	-
FHA 646	Air temperature	−20 to +80 °C	0.01	±0.4 °C
FHA 646	Air relative humidity	5 to 98%	0.1%	±2%
FYA 600	CO_2_	0 to 0.5%	0.001%	±0.01%
BEHA Unitest 93411 D	Sound level	30 to 135 dB	0.1 dB	±2 dB
Dust-Track™ II Aerosol Monitor 8530	Airborne dust concentrations	0.001 to 150 mg·m^−3^	0.001 mg·m^−3^	±0.1%
KERN-440-35N	Material mass	0 to 400 g	0.01 g	±0.01 g

**Table 4:** Results of measurements of the external thermal-humidity environment of outdoor air (temperature t_e_, °C; relative humidity RH_e_, %), wind speed (w_e_, m·s^−1^), and measured values of the indoor environment (temperature t_i_, °C; relative humidity RH_i_, %; concentration of CO_2i_, ppm; sound pressure level L_pAi_, dB) in stable during 7 days of the measurement. SD—standard deviation.

t_e_ ± SD	RH_e_ ± SD	w_e_ ± SD	t_i_ ± SD	RH_i_ ± SD	CO_2i_ ± SD	L_pAi_ ± SD
°C	%	m·s^−1^	°C	%	ppm	dB
10.0 ± 4.9	71.7 ± 13.7	2.2 ± 1.3	15.3 ± 2	65.4 ± 5.1	921.5 ± 374.5	47.0 ± 8.7

**Table 5:** Results of measurements of the external thermal-humidity environment of outdoor air (temperature t_e_, °C; relative humidity RH_e_, %), wind speed (w_e_, m·s^−1^), and measured values of the indoor environment (temperature t_i_, °C; relative humidity RH_i_, %; concentration of CO_2i_, ppm; sound pressure level L_pAi_, dB) in stable during a working day. SD—standard deviation.

t_e_ ± SD	RH_e_ ± SD	w_e_ ± SD	t_i_ ± SD	RH_i_ ± SD	CO_2i_ ± SD	L_pAi_ ± SD
°C	%	m·s^−1^	°C	%	ppm	dB
7.5 ± 3.5	74.8 ± 13.4	1.7 ± 0.8	14.0 ± 1.0	65.4 ± 4.3	966.4 ± 490.7	46.1 ± 8.5

**Table 6:** Results of measurements of the external thermal-humidity environment of outdoor air (temperature t_e_, °C; relative humidity RH_e_, %), wind speed (w_e_, m·s^−1^), and measured values of the indoor environment (temperature t_i_, °C; relative humidity RH_i_, %; concentration of CO_2i_, ppm; sound pressure level L_pAi_, dB) in stable during a non-working day.

t_e_ ± SD	RH_e_ ± SD	w_e_ ± SD	t_i_ ± SD	RH_i_ ± SD	CO_2i_ ± SD	L_pAi_ ± SD
°C	%	m·s^−1^	°C	%	ppm	dB
9.1 ± 1.0	81.7 ± 12.0	2.1 ± 0.9	15.8 ± 1.5	66.9 ± 2.8	836.2 ± 303.3	47.1 ± 8.8

SD—standard deviation.

**Table 7:** Average values and standard deviations of basic properties of bedding materials.

Properties of Materials	Straw	Mixture of Sawdust and Shavings 1:1
Bulk density, kg·m^−3^	27.06 ± 1.36	150.66 ± 11.13
Stem length, mm	130.4 ± 59.3	-
Water content, wet basis, %	7.83 ± 0.14	9.70 ± 0.15

**Table 8:** Number and percentages of smokers (up to 10 cigarettes per day), former smokers, and non-smokers; assignment of respondents to NoEx, LoEx, MedEx, and HiEx groups by weekly number of hours spent in equestrian facilities.

Smoking	NoEx (46 People) <1 h/Week	LoEx (20 People) 1–5 h/Week	MedEx (8 People) 5–10 h/Week	HiEx (20 People) >10 h/Week
Smokers	3	4	1	2
Former smokers	6	4	0	1
Non-smokers	37	12	7	17
Smokers (%)	7	20	13	10
Former smokers (%)	13	20	0	5
Non-smokers (%)	80	60	87	85

**Table 9:** Number and percentages of respondents who reported 0, 1, and 2-or-more respiratory diseases in previous anamnesis; assignment of respondents to NoEx, LoEx, MedEx, and HiEx groups by weekly number of hours spent in equestrian facilities.

Previous Medical History (in Numbers)	NoEx (46 People) <1 h/Week	LoEx (20 People) 1–5 h/Week	MedEx (8 People) 5–10 h/Week	HiEx (20 People) >10 h/Week
No disease	31	14	5	14
One disease	13	3	2	5
Two-or-more diseases	2	3	1	1
No disease (%)	68	70	62	70
One disease (%)	28	15	25	25
Two-or-more diseases (%)	4	15	13	5

**Table 10:** Number and percentages of respondents who reported asthma, allergies and chronic obstructive pulmonary disease (COPD) in previous anamnesis; assignment of respondents to NoEx, LoEx, MedEx, and HiEx groups by weekly number of hours spent in equestrian facilities.

Previous Medical History (Type of Disease)	NoEx (46 People) <1 h/Week	LoEx (20 People) 1–5 h/Week	MedEx (8 People) 5–10 h/Week	HiEx (20 People) >10 h/Week
Asthma	2	4	1	1
Allergies	15	4	3	6
COPD	0	2	0	0
Asthma (%)	4	20	13	5
Allergies (%)	33	20	38	30
COPD (%)	0	10	0	0

**Table 11:** Number of respondents reporting 0, 1, 2, 3, or 4 subjective respiratory problems (difficulties); assignment of respondents to NoEx, LoEx, MedEx, and HiEx groups by weekly number of hours spent in equestrian facilities.

Number of Reported Subjective Respiratory Problems	NoEx (46 People) <1 h/Week	LoEx (20 People) 1–5 h/Week	MedEx (8 People) 5–10 h/Week	HiEx (20 People) >10 h/Week
No problem	9	3	1	3
One problem	12	7	1	3
Two problems	13	2	4	8
Three problems	10	4	2	5
Four problems	2	4	0	1

**Table 12:** Type of reported subjective respiratory problems (difficulties such as allergic rhinitis, dry cough, moist cough, difficulty breathing, none of the above; more choices possible); assignment of respondents to NoEx, LoEx, MedEx, and HiEx groups by weekly number of hours spent in equestrian facilities.

Type of Reported Subjective Respiratory Problems	NoEx (46 People) <1 h/Week	LoEx (20 People) 1–5 h/Week	Medex (8 People) 5–10 h/Week	HiEx (20 People) >10 h/Week
(Allergic) rhinitis	27	8	5	12
Dry cough	24	13	4	15
Moist cough	9	5	2	5
Difficulty breathing	15	11	4	6
None of the above	9	3	1	3

**Table 13:** Results of measurements and statistical comparison of total dust mass concentration TDC (μg·m^−3^) and concentrations of fractions (μg·m^−3^) PM_10_, PM_4_, PM_2.5,_ and PM_1_ during various cleaning operations, sweeping and bedding in the stable, in the empty indoor arena without horses, and in the outdoor arena with horses. Different superscript letters (^a^, ^b^, ^c^, ^d^) are a sign of a highly significant difference (ANOVA; Tukey HSD test; *p* ≤ 0.01) between the measured values in the individual columns. SD—standard deviation.

Measured Object	TDC ± SD	PM_10_ ± SD	PM_4_ ± SD	PM_2.5_ ± SD	PM_1_ ± SD
A. Stable: cleaning dirt in boxes	111.8 ± 35.5 ^a^	45.9 ± 13.1 ^a^	31.3 ± 6.2 ^a^	24.9 ± 1.6 ^a^	17.6 ± 0.9 ^a^
B. Stable: straw bedding	3786.7 ± 2271.6 ^b^	877.2 ± 468.9 ^b^	416.0 ± 212.0 ^b^	54.4 ± 34.9 ^b^	22.6 ± 4.3 ^b^
C. Stable: bedding sawdust, shavings	2663.5 ± 1990.0 ^c,d^	877.5 ± 314.2 ^b^	236.8 ± 50.5 ^c^	69.2 ± 22.1 ^b^	21.6 ± 2.0 ^b^
D. Stable: cleaning center corridor	3186.2 ± 2306.8 ^b,d^	185.9 ± 105.4 ^c^	182.8 ± 61.1 ^d^	173.1 ± 50.3 ^c^	45.2 ± 12.2 ^c^
E. Indoor Arena: empty	20.3 ± 1.6 ^a^	20.1 ± 1.0 ^a^	18.7 ± 1.0 ^a^	17.8 ± 0.4 ^a^	14.5 ± 0.8 ^d^
F. Outdoor Arena: horses	476.0 ± 475.0 ^a^	44.6 ± 22.9 ^a^	25.2 ± 0.9 ^a^	25.1 ± 0.9 ^a^	19.6 ± 0.6 ^a,b^

**Table 14:** Results of noise measurement in the stable during the working day.

Operational Activity	Average L_pA_ ± SD dB	Minimum L_pA_ dB	Maximum L_pA_ dB
Horses resting	42.7 ± 6.3	33.3	59.9
Feeding	52.4 ± 7.9	36.9	62.0
Moving horses to paddock and back	63.0 ± 9.8	50.4	74.5
Cleaning the stable	52.3 ± 6.7	44.1	68.3
Teaching students	45.3 ± 7.7	35.7	60.9
Equestrian club	49.1 ± 8.2	37.9	61.2
Closing stable and staff departure	50.9 ± 5.6	45.3	56.5
All day 24 h	46.1 ± 8.5	33.3	74.5

SD—standard deviation.

**Table 15:** Results of noise measurement in the stable during the non-working day.

Operational Activity	Average L_pA_ ± SD dB	Minimum L_pA_ dB	Maximum L_pA_ dB
Horses resting	41.9 ± 4.1	35.6	56.9
Feeding	52.2 ± 6.3	45.6	63.4
Moving horses to the paddock and back	53.9 ± 1.0	52.9	55.3
Cleaning the stable	43.5 ± 4.6	37.4	53.6
Equestrian club	52.5 ± 11.4	41.8	86.0
Closing stable and staff departure	45.2 ± 1.0	44.2	46.1
All day 24 h	47.1 ± 8.8	35.6	86.0

SD—standard deviation.

**Table 16:** Limit values for the protection of human health to be attained by 1st January 2030 and 11th December 2026 [118].

PM	Averaging Period	Limit Value (μg·m^−3^)	Note	Year
PM_2.5_	1 day	25	not to be exceeded more than 18 times per calendar year	2030
	Calendar year	10	-	
PM_10_	1 day	45	not to be exceeded more than 18 times per calendar year	2030
	Calendar year	20	-	
PM_2.5_	Calendar year	25	-	2026
PM_10_	1 day	50	not to be exceeded more than 35 times per calendar year	2026
	Calendar year	40	-	

## Data Availability

The horse handlers and riders gave consent for the raw data to be used by the researcher only, but any queries involving the processing of these data can be directed to the corresponding author.
